# Molecular and epigenetic analysis of the fragile histidine triad tumour suppressor gene in equine sarcoids

**DOI:** 10.1186/1746-6148-8-30

**Published:** 2012-03-16

**Authors:** Maria Strazzullo, Annunziata Corteggio, Gennaro Altamura, Romina Francioso, Franco Roperto, Maurizio D'Esposito, Giuseppe Borzacchiello

**Affiliations:** 1 Institute for Animal Production System in Mediterranean Environment, National Research Council, Via Argine, 1085, 80147 Naples, Italy; 2 Department of Pathology and Animal Health, University of Naples Federico II, Via Veterinaria, 1, 80137 Naples, Italy; 3 Institute of Genetic and Biophysics ABT, National Research Council, Via P. Castellino 111, 80131 Naples, Italy; 4 IRCCS Neuromed, Pozzilli, Italy

## Abstract

**Background:**

Sarcoids are peculiar equine benign tumours. Their onset is associated with Bovine Papillomavirus type -1 or -2 (BPV-1/2) infection. Little is known about the molecular interplay between viral infection and neoplastic transformation. The data regarding papillomavirus infections in human species show the inactivation of a number of tumour suppressor genes as basic mechanism of transformation. In this study the putative role of the tumour suppressor gene Fragile Histidine Triad (FHIT) in sarcoid tumour was investigated in different experimental models. The expression of the oncosuppressor protein was assessed in normal and sarcoid cells and tissue.

**Results:**

Nine paraffin embedded sarcoids and sarcoid derived cell lines were analysed for the expression of FHIT protein by immunohistochemistry, immunofluorescence techniques and western blotting. These analyses revealed the absence of signal in seven out of nine sarcoids. The two sarcoid derived cell lines too showed a reduced signal of the protein. To investigate the causes of the altered protein expression, the samples were analysed for the DNA methylation profile of the CpG island associated with the FHIT promoter. The analysis of the 32 CpGs encompassing the region of interest showed no significative differential methylation profile between pathological tissues and cell lines and their normal counterparts.

**Conclusion:**

This study represent a further evidence of the role of a tumour suppressor gene in equine sarcoids and approaches the epigenetic regulation in this well known equine neoplasm. The data obtained in sarcoid tissues and sarcoid derived cell lines suggest that also in horse, as in humans, there is a possible involvement of the tumour suppressor FHIT gene in BPV induced tumours. DNA methylation seems not to be involved in the gene expression alteration. Further studies are needed to understand the basic molecular mechanisms involved in reduced FHIT expression.

## Background

Sarcoids are benign tumours of fibroblastic origin affecting the skin of horses, mules and donkeys and are considered to be the most common equine cutaneous neoplasm worldwide [1]. They are histologically characterized by disorganized dermal proliferation of spindle-shaped fibroblasts that form whorls and by epidermal hyperplasia, hyperkeratosis, and rete peg formation [2,3]. The tumours most frequently arise from the skin of the head, ventral abdomen, legs and the paragenital region [4]. Sarcoids are locally invasive and often occur at sites of previous injury or scarring; additionally, they very rarely regress, more often persist and can be locally aggressive. Sarcoids may exist as single or multiple lesions and six clinical types are recognized: occult, verrucous, nodular, fibroblastic, mixed and malignant [5]. Currently there is no effective therapy available for sarcoids [6].

BPV-1 and less commonly BPV-2 infection is now recognized as one of the etiological factors of sarcoids. However, the pathology of this common equine dermatological neoplasm is not completely understood. Recent studies have highlighted the role of the BPV oncogenes E5 and E7 in the carcinogenesis [7-9], but little is known about the molecular interplay between viral infection and neoplastic transformation.

Different Papillomaviruses (PVs), such as Human papillomavirus type 16 (HPV-16) and BPV-1, seems to share common processes of infection [10]. Several studies associate the development of cancer to the loss of function of a number of tumour suppressor genes. FHIT is a well characterized tumour suppressor gene involved in the neoplastic transformation associated to the PVs infection [11].

The FHIT gene encodes a protein of 147 aa, a diadenosine triphosphate hydrolase that cleaves the diadenosine substrate into adenosine diphosphate (ADP) and adenosine monophosphate (AMP) [12], its physical location overlaps with the FRA3B locus, the most active human common fragile site [13]. FHIT protein is ubiquitously expressed in human tissues. Interestingly this protein is directly involved in tumour suppression independently of its hydrolytic activity. It may function as intracellular and extracellular signalling molecules by interacting with specific proteins involved in the regulation of proliferative and apoptotic cellular processes [14-16]. In a variable percentage of cases, depending on the tumour type, FHIT loss of function is determined by the hypermethylation status of the associated CpG island with the consequent repression of gene transcription [11]. While alterations in the FHIT gene have been reported in several human cancers, the putative role of this tumour suppressor gene and its epigenetic alteration in animal tumours are not well known [17,18]. In order to obtain new insights into BPV-mediated carcinogenesis, we investigated the status of FHIT protein expression in equine sarcoid cell lines and sarcoid tumours. Subsequentely, the DNA methylation status of equine FHIT CpG island spanning the putative promoter was investigated.

## Results

### FHIT protein expression in sarcoids and cell lines

**Figure 1:**
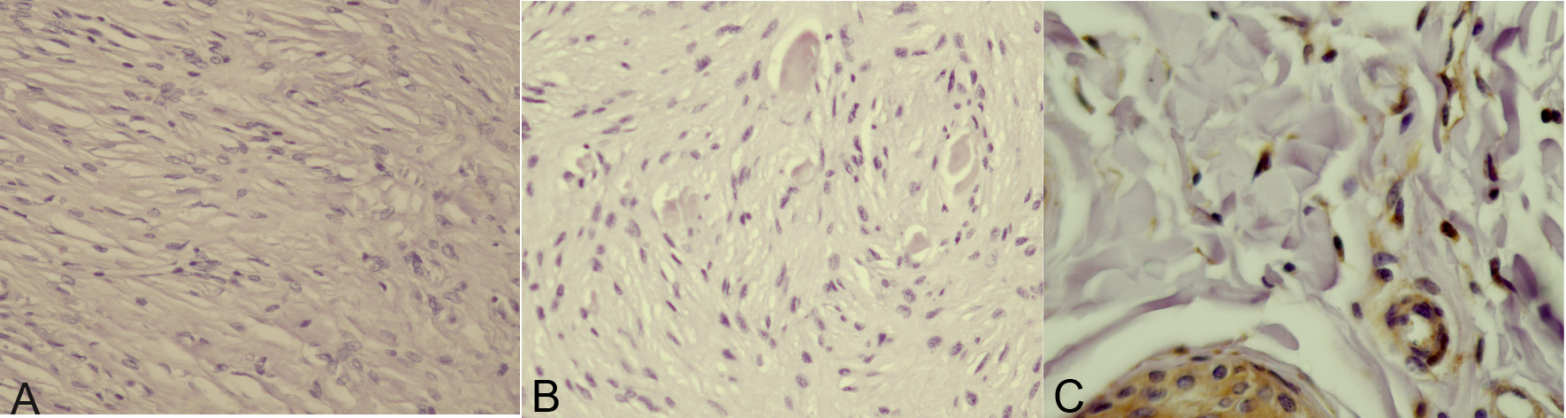
**Expression of FHIT in equine sarcoids and normal skin**. A) FHIT protein is not expressed in neoplastic fibroblasts. Streptavidin-biotin peroxidase method. Mayer's haematoxylin nuclear counterstaining. × 120. B) Neoplastic fibroblasts do not express FHIT. Streptavidin-biotin peroxidase method. Mayer's haematoxylin nuclear counterstaining. × 120. C) FHIT is expressed in normal skin. Normal fibroblast display cytoplasmic immunosignal. Normal blood vessels and sweat glands also stain. Streptavidin-biotin peroxidase method. Mayer's haematoxylin nuclear counterstaining. × 240.

To gain insights into the molecular mechanisms of fibroblast transformation in equine sarcoid, we analyzed tumour samples and sarcoid derived cell lines for the expression of the FHIT protein. The anti-FHIT polyclonal antibody was used to stain histological sections of 9 BPV positive tumour samples and 1 normal skin sample. No staining for FHIT protein was observed in 7 out of 9 tumour cases (80%) (Figure 1A, B). Only 2 out of the 9 (20%) sarcoid samples, showed weak intracytoplasmic immunosignal for FHIT. Fibroblasts of normal skin sample displayed nuclear and cytoplasmic staining. Normal blood vessels and sweat glands also stained (Figure 1C).

**Figure 2:**
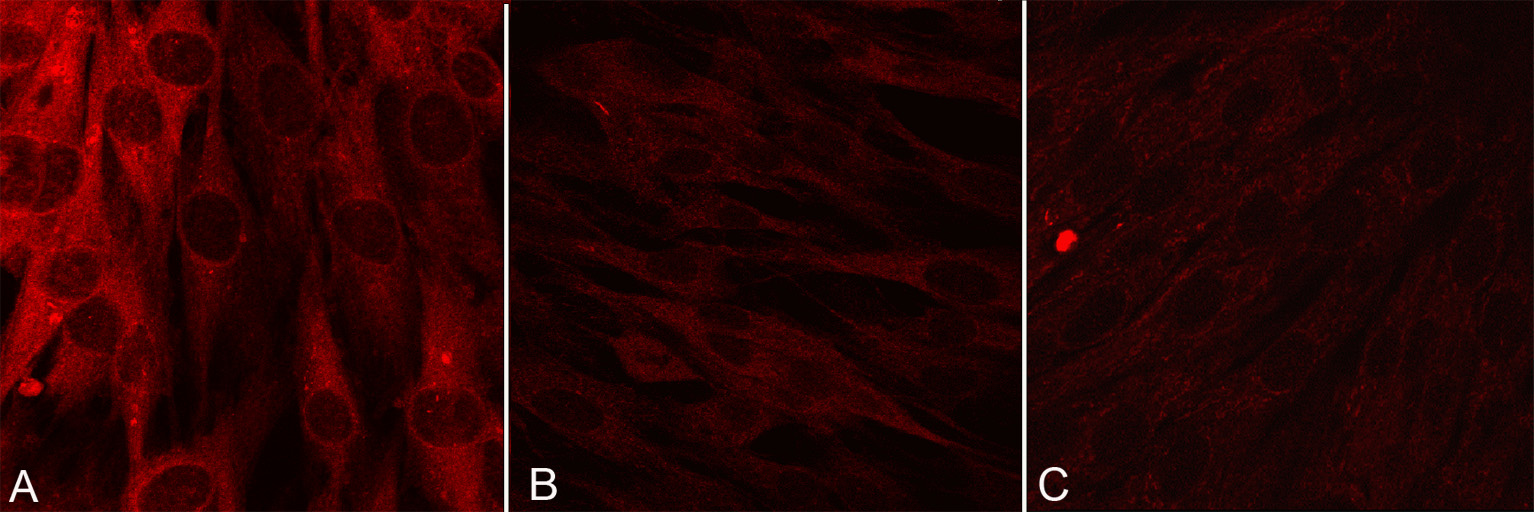
**Immunofluorescence-mediated detection of FHIT protein in E-DERM and sarcoid derived cell lines**. A) E-DERM normal fibroblasts show cytoplasmic staining X120. B) Fully transformed fibroblasts from EqSO1a show faint cytoplasmic staining X120. C) Fully transformed fibroblasts from EqSO4b show a very faint cytoplasmic staining. X120.

**Figure 3:**
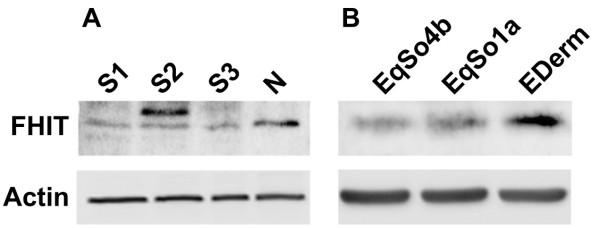
**Western blotting analysis of FHIT protein in sarcoid tissues and sarcoid derived cell lines**. A) The expression of FHIT is reduced in sarcoids (S1, S2 and S3) compared to normal skin sample (N). B) FHIT protein expression in EqSO1a and EqSO4b in comparison with E-DERM. The lower blot shows actin expression to demonstrate the same amount of protein loaded on the gel.

We also analyzed three equine fibroblast cell lines for the expression of FHIT protein, one normal and two derived from sarcoids. By indirect immunofluorescence, distinct cytoplasmic staining was detected in the normal equine fibroblast cell line (E-DERM). The immunosignal was detected only within the cytosol and the pattern was diffuse. The sarcoid cell lines EqSO1a and EqSO4b showed very weak cytoplasmic immunofluorescence signal for FHIT protein as compared with E-DERM (Figure 2). However, EqSO4b cells showed a weaker immunofluorescence staining than EqSO1a. To further confirm the lack of FHIT protein expression in tumours, three sarcoid samples, one skin sample from a healthy horse and the cell lines were analysed by immunoblotting. The anti-FHIT antibody recognized a band of the expected molecular weight in the neoplastic tissues, normal skin and all fibroblast cell lines. An increase in the amount of FHIT protein level in non-neoplastic skin versus tumour samples was observed (Figure 3A); moreover FHIT expression levels were lower in sarcoid cell lines compared to E-DERM (Figure 3B).

The immunohistochemical and biochemical data indicate that the expression of the FHIT protein is down-regulated in tumour samples and in fully transformed sarcoid fibroblast lines.

### Equine FHIT locus and transcript characterization

GenBank search for equine FHIT gene sequences gave the predicted coding sequence from the ATG to the stop codon [XM_001490610]. To obtain the putative regulatory region sequence of the equine FHIT locus, the human FHIT CpG island sequence [U76263] was used to query genomic databases. This sequence encompasses the region of the non coding exon 1 and the partial intron 1 and represents the region analysed in human cancers for differential methylation profile. The U76263 human sequence was compared, through the BLAT algorithm of the UCSC genome browser, with the Horse Sep. 2007 (Broad/equCab2) Assembly.

**Figure 4:**
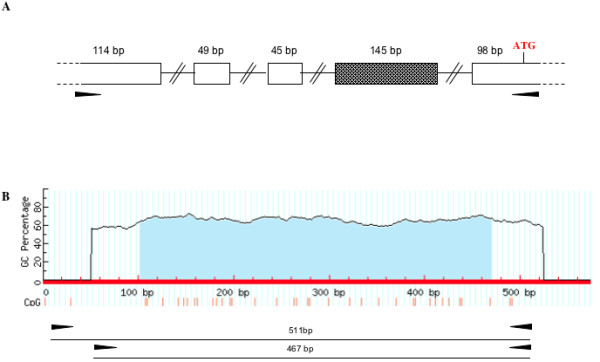
**Equine FHIT genomic locus organization**. A) The organization of the 5'UTR of the FHIT transcript is shown; the shaded box represent the exon present only in the isoform 2. B) The FHIT CpG island is reported according the MethPrimer software scheme and the bisulfite primer positions are also shown.

The region (573 bp) showed 87% similarity with the human sequence. The correct localization of the CpG island and the co-linearity of the UTR sequence with the FHIT coding sequence were confirmed through direct sequencing of PCR fragments obtained using respectively genomic DNA and cDNA as templates. These analyses were performed in E-DERM cell line. The analyses of the PCR fragments amplified using cDNA as template, showed the presence of two different transcripts. The analysis of these sequences through the BLAT algorithm revealed the presence of 4 exons in isoform 1 and 5 exons in isoform 2. The additional exon of isoform 2 (exon 4) is 145 bp long. Figure 4A shows the 5' FHIT transcript organisation in the horse genome. The equine FHIT 5'UTR region organization is comparable with the data deriving from other species such as *Bos *[17]. The DNA sequences of both transcripts have been deposited in GenBank [JN403048, JN403049].

### Equine FHIT CpG island methylation analysis in sarcoid tissues and cell lines

Sarcoid samples were investigated for the DNA methylation profile of the CpG island associated to the putative regulative region.

The CpG island sequence includes 32 CpG dinucleotides, and the region analysed corresponds to the CpG island predicted with the CpGplot analysis. It is slightly larger then the region analysed in human and bovine tumours [17]. Figure 4B shows the equine CpG island and the position of the amplification primers obtained through the MethPrimer algorithm. The bisulfite DNA conversion and the subsequent cloning and sequencing analysis were performed also for a BPV negative normal skin sample. For all samples at least 10 clones were sequenced. The extensive analysis of all CpG dinucleotides present in the region, showed only some random differentially methylated position in comparison with the normal control (Additional file 1: Figure S1). Similar results were also obtained extending the DNA methylation analysis to the sarcoid cell lines, EqSO1a and EqSO4b compared to the E-DERM fibroblast cell lines.

## Discussion

Sarcoids account for an high percentage of all equine neoplasms and are the most common tumour diagnosed in horses [1]. Few studies have been carried out so far concerning the molecular mechanisms underlying equine sarcoids carcinogenesis. It is well known that BPV is involved in the pathogenesis of the sarcoid, and the role of its oncoproteins in the carcinogenic process has been recently clarified [19,20], however little is known about the molecular relationship between viral infection and neoplastic transformation. Recently, Yuan et al. [21], have described the upregulation of MMP-1 and AP1 in sarcoid, underlying the invasivity potential of such tumour. To identify the key molecules in these phenomena and on the basis of the data deriving from other species, we analysed the role of the tumour suppressor FHIT gene. FHIT is a tumour suppressor gene and many lines of evidence support the association between HPV infection and FHIT expression alteration in different cancer types [22,23]. However, FHIT downregulation has been recorded also in BPV induced bovine urinary bladder tumours which are composed of both epithelial and vascular (i.e. mesenchymal) tumours (personal observations).

In this study we have characterized the expression of FHIT protein by examining it in a normal fibroblast cell line (E-DERM) and fully transformed sarcoid fibroblast lines explanted from equine sarcoid tumours (EqSO1a and EqSO4b), as well as in normal skin and sarcoid tissues. The data we obtained document, for the first time, a reduction of FHIT protein expression in in vitro model of sarcoid-derived cell lines as well as in naturally occurring tumours. The consistency of data between in vivo and in vitro systems strenghten the validity of such cell culture system for gene expression analysis.

Our data indicate that FHIT protein is expressed in normal fibroblasts, whereas it is much reduced in both in vivo and in vitro tumours. It is worthwhile noting that EqSO4b has lower level of the protein compared to the EqSO1a. The EqSO4b cell line contains higher levels of viral genome and viral oncoprotein transcripts than EqSO1a [24]. We may speculate that altered expression of FHIT protein may correlate with viral load and/or viral oncogenes expression. However, further studies are needed to clarify this point. Sarcoids may exist as six different clinical types [5]; although we have examined a limited number of samples, we suggest a lack of correlation between down-regulation of FHIT expression and clinical appearance, indicating a common mechanism underlying the reduction of protein expression acting early during the development of equine sarcoids.

Our results are in agreement with several previous studies indicating that the loss of FHIT expression is frequent in a variety of tumors [25,26] above all in PVs induced cervical lesions [11,22,27,28]. Lowered levels of the FHIT protein were also documented in pagetoid variant of BPV induced urothelial tumour [18].

Promoter methylation is the primary epigenetic alteration associated with transcriptional silencing of tumour suppressor genes during tumorigenesis [29-32]. To determine the reasons for diminished FHIT expression in sarcoid tumour we performed the analysis of the methylation status of the FHIT 5'-CpG island. Our data indicated that FHIT gene promoter hypermethylation did not occur either in sarcoid tissues or in sarcoid derived cell lines, suggesting that this epigenetic mechanism may be not involved in FHIT aberrant expression in sarcoids. This is in accordance with a previous paper documenting no aberrant methylation causing FHIT promoter silencing in BPV induced vesical tumors of cattle [17]. It is reasonable to assume that BPV does not alter the methylation status of FHIT promoter. It is worth noting that similar data have been obtained both in vitro and in vivo, thus again validating the sarcoid derived cell lines as a good model for the study of sarcoids [24]. Our data show the presence of an alternative splicing of equine FHIT transcript in 5'UTR region; this is consistent with the data obtained in other species such as human and bovine [17], in which several different 5'UTR regions were described. A well-known regulatory role has been described for the 5' and 3' UTR region in mammalian genome [33]. The complex structure of the 5'UTR region of FHIT locus may suggest that this region might be target of post-transcriptional regulative mechanism.

In human species the mutational analysis of FHIT indicates the potential role of other mechanisms such as point mutation and deletions, with loss of heterozigosity (LOH). The human FHIT gene encompasses the FRA3B fragile site, a common target of genetic alterations [34]. This locus is a hot spot for early inactivation in carcinogen-exposed tissue [13,35]. FRA3B fragile site is also reported to be the site for HPV-16 integration and demonstrated to undergo frequent LOH in cervical carcinoma [36]. Different PVs, such as HPV-16 and BPV-1, seems to share common processes of infection [10]. We speculate that the involvement of BPV infection in equine tumorigenesis may be related with FHIT LOH, but this issue needs further investigation.

Our findings suggest an association between BPV infection and FHIT protein expression alteration raising the possibility of a mechanistic role for the FHIT gene as a cofactor with BPV in triggering the development of equine sarcoid tumour.

## Conclusions

Biochemical and immunohistochemical analyses suggest that FHIT protein is often reduced in sarcoid tissues and cell lines. The epigenetic analysis suggest that altered DNA methylation is not associated with the altered expression of the FHIT protein. While further studies are needed to clarify the molecular mechanisms for FHIT protein downregulation, we suggest that this tumour suppressor gene is an important player in sarcoid transformation.

## Methods

### Tumour Samples

Samples of equine sarcoid (N°9) were derived from the archives of the Department of Pathology and Animal Health, University of Naples "Federico II". Normal skin from healthy horses was also examined. Formalin-fixed paraffin embedded tissue was available from each case. Sections taken from these blocks and stained by Haematoxylin and eosin (HE) were re-evaluated to confirm the diagnosis. The 9 samples were derived from 9 different animals. All sarcoids were known to be positive for BPV DNA [9].

Sarcoids and normal skin, from the same animals, were also collected and immediately frozen at -80°C for biochemical analysis.

### Cell cultures

E-DERM fibroblast cell lines, derived from horse dermis, were obtained from the American Type Culture Collection. EqSO1a and EqSO4b sarcoid derived cell lines have been previously described [24]. All cells were maintained in culture in DMEM (Dulbecco's modified eagle medium) supplemented with 10% FBS (Gibco) in a 37°C humidified atmosphere of 5% CO2 in air.

### Immunohistochemistry

Nine sarcoid samples and one normal skin sample were stained. Briefly, paraffin sections were deparaffinized, and blocked for endogenous peroxidase in 0.3% H_2_O_2 _in methanol for 20 min. Antigen enhancement was performed by pretreating with microwave heating (twice for 5 min each at 525 W). The anti-FHIT antibody (Santa Cruz, USA) was applied at 1:100 dilution overnight at room temperature in a humified chamber. The slides were washed three times with phosphate-buffered saline (PBS), then incubated with appropriate secondary antobodies as previously reported [37]. Sections were washed three times with PBS and then incubated with streptavidin-conjugated to horseradish peroxidase (LSAB Kit; DakoCytomation, Denmark). Colour development was obtained by treatment with diaminobenzidine (DakoCytomation, Denmark) for 5 min. Sections were counterstained with Mayer's haematoxylin. In the corresponding negative control section, the primary antibodies were either omitted or replaced with appropriate normal sera.

The scoring of the immunoreactivity was determined in a 'blind' study by two observers (GB and AC). The intensity of labelling in each specimen was scored from absent to very strong immunosignal.

### Immunofluorescence and confocal laser-scanning microscopy

E-DERM, EqSO1a and EqSO4b cell lines were grown for 2 days on coverslips, washed with PBS, fixed in 4% paraformaldehyde for 20 min, permeabilized with 0,1% triton X-100 in PBS 5 min. The slides were blocked with 2% BSA for 30 min. The anti-FHIT primary antibody was applied O/N at 4°C in a humified chamber at 1:50 dilution and after washing with PBS, incubated with Alexa Fluor 546 goat anti-rabbit 30 min at RT (Molecular Probes. Leiden, The Netherlands).

Finally, after washing with PBS, the slides were mounted in aqueous medium PBS:Glycerol 1:1 (Sigma, Milan, Italy). For scanning and photography, a confocal laser-scanning microscope LSM-510 (Zeiss, Gottingen, Germany) was used. Alexa Fluor 546 was irradiated at 543 nm and detected with a 560 nm bandpass filter.

### Protein extraction and SDS PAGE/Western blotting

Three fresh tumour (S1, S2 and S3) and one sample of normal skin (N) were available for molecular analysis. These were snap frozen in liquid nitrogen and homogenized in ice-cold lysis buffer (50 mM Tris pH7.5; 150 mM NaCl; 1 mM EDTA; 0.25% Deoxicolic acid, 1% Triton X100) added with 20 mM sodium pyrophosphate, 0.1 mg/ml aprotinin, 2 mM phenylmethylsulphony fluoride (PMSF), 10 mM sodium orthovanadate (Na_2_VO_3_), and 50 mM NaF.

Cell lines were grown for 2 days in 60-mm dishes, washed with ice-cold phosphate saline buffer two times and lysed for 20 minutes in ice-cold lysis buffer. Tissue homogenates and cell lysates were clarified by centrifugation, and the quantity of proteins was determined by use of a protein assay kit (Bio-Rad Laboratories, Milan, Italy). 50 μg of total protein were boiled and fractionated in 15% SDS-PAGE gel. The proteins were blotted from the gel onto nitrocellulose membranes. The membranes were blocked with 5% non-fat dry milk in TBS buffer at room temperature, washed with TBS-0.1% Tween and incubated with anti-FHIT antibody at dilution 1: 500 (Santa Cruz). After appropriate washing steps, peroxidase-conjugated anti-rabbit IgG (Amersham Pharmacia Biotech) were applied for 1 hour at 1:5,000. After washing, bound antibody was visualized on ECL film (Amersham Pharmacia Biotech). The blots were stripped and reprobed against mouse anti-actin antibody (Calbiochem) at 1:5,000 to confirm equal loading of proteins in each lane.

### Bioinformatic web tools for sequence analyses and primer design

NCBI Genbank http://www.ncbi.nlm.nih.gov/sites/entrez to obtain specific sequence; UCSC BLAT http://genome.ucsc.edu/cgi-bin/hgBlat to obtain the sequence of non-coding regions and to recovery and compare the sequences of different species. BLAST algorithm http://blast.ncbi.nlm.nih.gov/ to identify the more informative sequences, to compare the horse specific nucleotidic sequences with that of other species; EMBOSS CpGPlot/CpGReport/Isochore http://www.ebi.ac.uk/Tools/emboss/cpgplot/ for the analysis of the CpG content; MethPrimer to design primers for methylation analysis http://www.urogene.org/methprimer/index1.html; Primer3 http://primer3.sourceforge.net/ and NCBI primer-blast http://www.ncbi.nlm.nih.gov/tools/primer-blast/index.cgi to design PCR primers.

### Cell lines DNA and RNA extraction

Genomic DNA was extracted from one 60-mm dish respectively of E-DERM, EqSO1a and EqSO4b cell lines according to the Wizard Genomic DNA Purification Kit protocol (Promega Corporation, USA). The purified DNA was resuspended in TE.

Total RNA was extracted from E-DERM, EqSO1a and EqSO4b cell lines. The cell pellet derived from two 60-mm dishes, cultured as described above, was resuspended in 1 ml of Trizol (Invitrogen, Carlsbad, CA, USA). The RNA purification was obtained according to the Trizol protocol.

### PCR and RT-PCR analyses

The equine specific FHIT CpG island was obtained by genomic DNA PCR amplification with the following primers: FHITCpGF TCCTGAGAGGGACAGTGGTT, FHITCpGR GGGGAGGGTTAGGGTGAG. The purified PCR product (573 bp) was directly sequenced by the dye terminator method (PRIMM Facility, Naples, Italy).

For all cell lines, cDNA was prepared, after DNAaseI RNAase free digestion, using 1 μg of total RNA according to the protocol of Quantitect Reverse Transcription for RT-PCR (Qiagen, Germany). The final volume was 20 μl. cDNA quality was tested through the amplification of the housekeeping gene GAPDH. The PCR primer sequences were GAPDHF GCCATCACCATCTTCCAG and GAPDHR GTTCACGCCCATCACAAAC and the PCR product lenght was 192 bp. The FHIT specific primers used were the following: FHITF2 GTCGGGAATTGTAGTCCTC, FHITF3 CTCTCTTCCCGGGTCTGTAA and FHITR2 GTTCACGAGGGCAAAGGATA. The F2 R2 product lenght was 359 bp; the F3 R3 product lenght was 451 bp. The purified PCR products were directly sequenced by the dye terminator method (PRIMM Facility, Naples, Italy).

### DNA extraction from paraffin-embedded sarcoid samples, bisulfite conversion and sequencing

Genomic DNA was isolated from paraffin-embedded sections using standard procedures and was precipitated with 250 mM NaCl and isopropanol. Extracted DNA (0.25 - 1 μg) was bisulfite-converted using the EpiTect Bisulfite Kit (Qiagen, Germany), according to the protocol relative to the paraffin embedded samples. Samples were eluted in a final volume of 20 μl. To study the differential methylation status of FHIT putative promoter, bisulfite sequencing was performed. Briefly, 1 μl of bisulfite-converted DNA was amplified using a double step PCR (semi-nested PCR) carried out using Gradient PCR Express (Hybaid, Middlesex, UK). Step 1 primer sequences are: bisFHITF1 TTTTTTGAGAGGGATAGTGGTTTT and bisFHITR1 ACCAATACTCAAAAATAAACCCTCA; step 2 primer sequences are: bisFHITF2 GTTATTATGGTTTTTGATTGGGTTG and the above cited bisFHITR1. The second amplification was carried out using 1 μl of first amplification product as template. The thermal cycling conditions were: 95°C for 3 min, followed by 95°C for 30 sec, 52°C for 45 sec and 72°C for 45 sec for 25 (first amplification) or 35 (second amplification) cycles. After amplification, PCR products were recovered from agarose gel using QIAquick Gel Extraction Kit (Qiagen), following manufacturer's instructions. Purified PCR products were cloned in the pGEMeasy cloning vector (Promega Corporation, USA). After cloning, DNA fragments were sequenced by the dye terminator method (PRIMM Facility, Naples, Italy).

## Authors' contributions

AC and MS have contributed equally to this paper. AC and GA have contributed to experimental design and drafted the manuscript. FR has contributed to interpretation of data. GB has conceived the study, coordinated the group and drafted the manuscript. MS has contributed to experimental design and drafted the manuscript. RF has contributed to performing experimental procedures. MDE has coordinated the group and drafted the manuscript. All authors read and approved the final manuscript.

## Supplementary Material

Additional file 1 **Figure S1**. **Bisulfite sequencing of equine FHIT CpG island**. In the top are summerised the CpG island (boxed) and the position of primers of the nested PCR. Circles below indicate methylation status: filled circles, methylated CpGs; open circles: unmethylated CpGs. Each row of circles corresponds to one clone. The number on top of circle is referred to CpG position and the column on the left is referred to number of indipendent clones sequenced. Click here for file
