# Supplementary figures and images for: Molecular and epigenetic analysis of the fragile histidine triad tumour suppressor gene in equine sarcoids
Source: BMC Vet Res. 2012 Mar 16;8:30. doi: 10.1186/1746-6148-8-30 (PMC3361464; doi:10.1186/1746-6148-8-30)

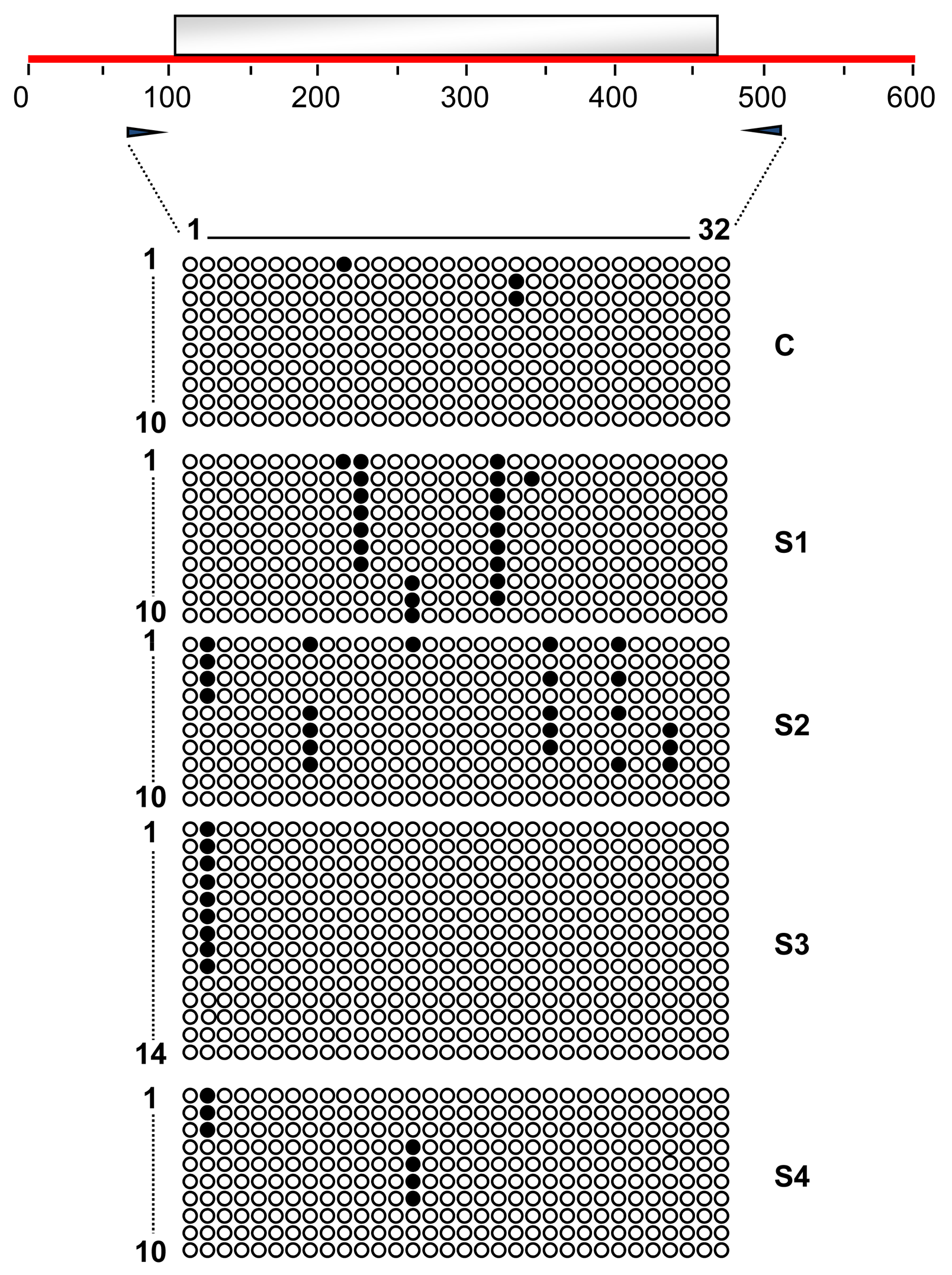

Supplement: Additional file 1 — Figure S1. Bisulfite sequencing of equine FHIT CpG island. In the top are summerised the CpG island (boxed) and the position of primers of the nested PCR. Circles below indicate methylation status: filled circles, methylated CpGs; open circles: unmethylated CpGs. Each row of circles corresponds to one clone. The number on top of circle is referred to CpG position and the column on the left is referred to number of indipendent clones sequenced. [file 1746-6148-8-30-S1.TIFF]
